# New composite thixotropic hydrogel composed of a polymer hydrogelator and a nanosheet

**DOI:** 10.1098/rsos.171117

**Published:** 2017-12-13

**Authors:** Yutaka Ohsedo, Masashi Oono, Kowichiro Saruhashi, Hisayuki Watanabe, Nobuyoshi Miyamoto

**Affiliations:** 1 Department of Life, Environment and Materials Science, Fukuoka Institute of Technology, 3-30-1, Wajiro-Higashi, Higashi-ku, Fukuoka 811-0295, Japan; 2 Comprehensive Research Organization, Fukuoka Institute of Technology, 3-30-1, Wajiro-Higashi, Higashi-ku, Fukuoka 811-0295, Japan; 3 Global Innovation Center, Kyushu University, 6-1 Kasuga-koen Kasuga-city, Fukuoka 816-8580, Japan; 4 Nissan Chemical Industries, Ltd., 2-10-1 Tsuboinishi, Funabashi, Chiba 274-8507, Japan

**Keywords:** molecular gel, composite, polymer hydrogelator, nanosheet

## Abstract

A composite gel composed of a water-soluble aromatic polyamide hydrogelator and the nanosheet Laponite®, a synthetic layered silicate, was produced and found to exhibit thixotropic behaviour. Whereas the composite gel contains the gelator at the same concentration as the molecular gel made by the gelator only, the composite gel becomes a softer thixotropic gel compared to the molecular gel made by the gelator only. The reason for this could be that bundles of polymer gelator may be loosened and the density of the polymer network increased in the presence of Laponite.

## 1. Introduction

Molecular gels [1–10] which are constructed from crystalline, fibrous networks of self-assembled, low-molecular weight gelator (LMWG) molecules have become a growing research area in materials chemistry. This is due to their easy preparation and the simple relationship between their well-defined chemical structure and their material properties, which aids the creation of functional materials for versatile applications such as medicine [11–13] and optoelectronics [14,15]. In the present decade, multi-responsiveness [16–22] and thixotropy [23,24] (mechanical gel- to-sol transition and subsequent sol-to-gel recovery after resting) of molecular gels [25–30] have received attention due to both development of new molecular gel functions and increased understanding of the physical chemistry of molecular gels. In particular, improving the thixotropic properties of molecular gels might help improve the poor mechanical properties of such gels. To develop a method for the production of thixotropic molecular gels, a new, simple strategy is required. For the purposes of developing a new, simple method for the production of thixotropic molecular gels, the mixing enhancement of gels and gelators was vigorously studied for finely designed, but intricate gelator systems [31–41]. Upon developing the mixing method, we studied simple mixing of organogels [42–47], hydrogels [48] with different alkyl chains and hydrogen bonding moieties [42–48], and simple mixing of LMWG and the inorganic nanosheet [49] Laponite®, a commercially available inorganic silicate rheology modifier, which is a type of inorganic nanosheet that exists in the form of discs (diameter, 30 mm; thickness, 1 nm) [50,51]. Through our studies, we showed that mixing induced thixotropy in the mixed molecular gels and the LMWG/Laponite composite molecular gel [49]. This may have been due to the improvement of network quality in the presence of Laponite by reducing bundle aggregation of network fibres. Following our creation of a thixotropic LMWG/inorganic nanosheet composite hydrogel, we attempted to broaden the application of this simple mixing method by mixing a polymer gelator [52–60] with the inorganic nanosheet. The aim of this was to create new thixotropic organic/inorganic composite molecular gels. This simple mixing method and the new thixotropic molecular gels obtained will be applicable to the production of medicinal materials.

**Scheme 1.**
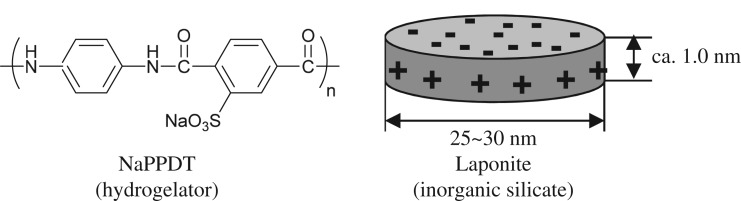
Chemical structure of NaPPDT and schematic illustration of Laponite.

Herein, we report a new, simple method for creating molecular hydrogels through the formation of a composite of a water-soluble aromatic polyamide, poly(3-sodium sulfo-*p*-phenylene terephthalamide) (NaPPDT) [61–65], which was found to form lyotropic liquid crystal hydrogels in aqueous solution [66,67], and Laponite (scheme 1). Although several systems that promote the mixing-induced enhancement of gel properties of mixed gel formulations, including organic/inorganic composite hydrogels, have been studied [68–73], our approach involved simple mixing of Laponite with a polymeric hydrogelator. Moreover, while polymer/nanosheet composites have been vigorously studied [74,75], no combination between a synthetic gelator and an inorganic nanosheet has yet been reported for such a thixotropic gel except for our previous report on a LMWG/ inorganic nanosheet system. In relation to this study, facile preparation processes of magnetic composite polymer hydrogels composed of iron oxide particles and poly(vinyl alcohol) have been reported in the literature [76–78].

## 2. Material and methods

### 2.1. Materials

NaPPDT was synthesized as shown in the literature [66]. Laponite® XLG (Rockwood Additives Limited) was provided by Wilbur-Ellis Co., (JAPAN) LTD. and used as received. Water was deionized with an Elix UV 3 Milli-Q integral water purification system (Nihon Millipore K.K).

The composite hydrogels were prepared by mixing NaPPDT aqueous solution or hydrogel with Laponite aqueous solution at room temperature as follows. Pale, yellow, solid NaPPDT was placed in a vial (mighty vial, no. 01; Maruemu Corporation) containing water at a specific concentration (wt%), and the vial was capped with a rubber seal. The vial was then heated in a dry bath at 90°C until the NaPPDT solid had dissolved, at this point the NaPPDT aqueous solution was left to stand for 1 h at room temperature. To this NaPPDT (aqueous or gel), Laponite aqueous solution (0.5–2.0 wt%) was added and mixed using a vortex genie (Scientific Industries, Inc) at room temperature. The mixed composite was left for 3 h before measurements.

### 2.2. Methods

The gelation tests were performed using a vial inversion method. Gelation was determined by visual observation after inversion of the vial. If the mixture did not drop at the vial inversion, the mixture was considered to have formed a gel.

The hydrogel was observed using a Leica DM2500 (Leica Microsystems) polarized optical microscope under crossed-Nicols.

Thixotropic behaviour was evaluated using the vial inversion method. The prepared hydrogel in the vial was shaken and mechanically collapsed using a vortex genie (Scientific Industries, Inc) for several seconds. The obtained sol was then allowed to set for a given time at room temperature, and the recovery of the gel state from the sol state was determined by visual observation after inversion of the vial.

Scanning electron microscopy (SEM) images were recorded using an SU-8000 scanning electron microscope (Hitachi High-Technologies Corporation) at 1.0 kV; the SEM sample (xerogel of hydrogel) was freeze-dried and placed on a conductive tape on the SEM sample stage. Pt, as a conductive material, was used as a coating (10 nm thick) on the sample.

Rheological measurements of the frequency sweep were performed at 25°C using an MCR-301 rheometer (Anton Paar Japan K.K.) with a parallel plate (8 mm diameter) at a gap of 0.50 mm and *γ* of 0.01%. Rheological measurements of the strain sweep were performed at 25°C using an MCR-301 rheometer with a parallel plate (8 mm diameter) at a gap of 0.50 mm and a constant angular frequency of 1 rad s^−1^. For the rheological measurements, the hydrogel sample was applied onto the parallel plate and sample stage and then any overflow gel was swept away. Step-shear measurements were carried out by repeatedly applying a normal strain (strain amplitude 0.01% and frequency 1 Hz) and a large strain (shear rate 3000 s^−1^ for 0.1 s).

The test of thixotropic hysteresis loop of the hydrogels was conducted with 1st cycle: 0.001 s^−1^ ∼ 5.0 s^−1^ ∼ 0.001 s^−1^ (120 s), 2nd cycle: 0.001 s^−1^ ∼ 20 s^−1^ ∼ 0.001 s^−1^ (120 s), 3rd cycle: 0.001 s^−1^ ∼ 100 s^−1^ ∼ 0.001 s^−1^ (120 s), 4th cycle: 0.001 s^−1^ ∼ 400 s^−1^ ∼ 0.001 s^−1^ (120 s), with 1 min intervals between cycles.

Infrared spectroscopy was performed using an FT/IR-620 (JASCO Corporation, resolution 0.25 cm^−1^) and the single reflection ATR method (ZnSe prism, 64 scans).

Small angle X-ray scattering (SAXS) data were recorded on a D8 Discover X-ray diffractometer (Bruker AXS K.K.) using CuK*α* at 26°C (the sample was placed in a 2 mm-diameter quartz glass capillary tube).

## 3. Results and discussion

### 3.1. Results

**Figure 1.**
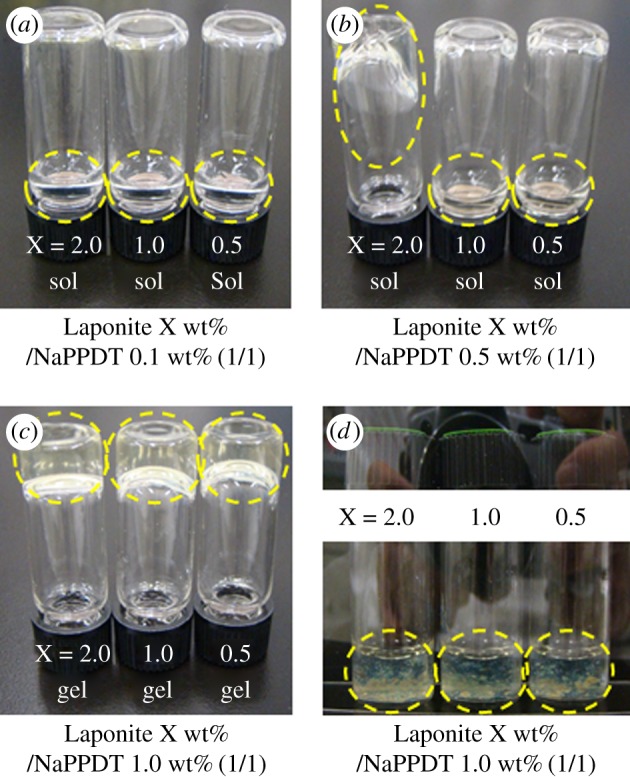
Gelation ability of the Laponite/NaPPDT (1/1, w/w) composite aqueous solutions: (*a*) Laponite aq. with 0.1 w% NaPPDT aq.; (*b*) Laponite aq. with 0.5% NaPPDT aq.; (*c*) Laponite aq. with 1.0 wt% NaPPDT hydrogel (NaPPDT formed a gel at 1.0 wt% aq.); and (*d*) (*c*) under crossed-Nicols with polarizing films.

Initially, we examined the gelation ability of mixed systems containing NaPPDT (Mn = 10 000), which was prepared as described in our literature [66], and Laponite aqueous solution. The composites were prepared by mixing NaPPDT aqueous solution or hydrogel with Laponite aqueous solution at room temperature (the critical gel concentration of NaPPDT in water is 1.0 wt% [66]). As shown in figure 1, Laponite X wt% (X = 2.0–0.5)/NaPPDT 1.0 wt% (1/1, w/w) formed hydrogels. The concentration of 1.0 wt% NaPPDT seemed to be essential for composite hydrogel formation. Although Laponite and NaPPDT did not form gels below 2.0 wt% and 1.0 wt%, respectively, they did form the composite hydrogel below these concentrations. This indicates that the mixing of Laponite and NaPPDT enhanced the gelation ability of each component, and that mixing-induced enhancement of the composite hydrogels occurred in this system. Notably, the composite gels maintained their gel state for at least six months.

Photographic and optical micrographic observations of the Laponite/NaPPDT hydrogels under crossed-Nicols revealed that the composite hydrogels retained the anisotropic nature that was observed in the NaPPDT hydrogels (figure 1*d* and electronic supplementary material, figure S1). This indicates that mixing of the Laponite (with no liquid crystallinity at 2.0 wt% aq., electronic supplementary material, figure S1) into true lyotropic NaPPDT hydrogel did not disrupt the anisotropic nature of NaPPDT.

**Figure 2.**
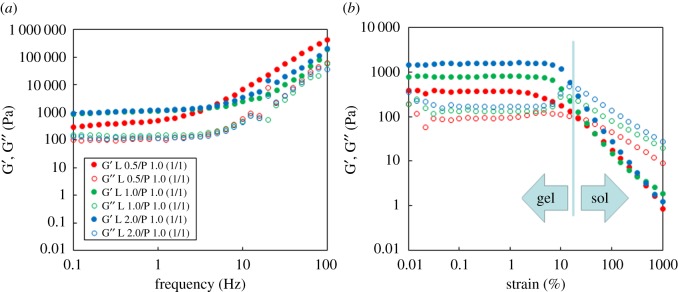
Dynamic rheological properties of the Laponite/NaPPDT composite hydrogels (denoted as L wt%/ P wt% (1/1, w/w)). (*a*) Frequency sweep and (*b*) strain sweep.

To investigate the gel state of the Laponite/NaPPDT composite hydrogel, rheometric measurements were examined (figure 2). The rheometric characteristics of the hydrogels confirmed the existence of a gel state and indicated that with increasing stress, the magnitudes of the storage modulus, *G*′, and the loss modulus, *G*′′, showed the transition of the gel state (*G*′ > *G*′′) to the sol state (*G*′ < *G*′′) [79]. In the frequency sweep, gelation is evidenced by the pseudo-plateau, where the magnitude of *G*′ is greater than that of *G*′′. In addition, the results show that the composite hydrogel became softer than the NaPPDT hydrogel at the same concentration probably because of the addition of the Laponite component (*G*′ for Laponite 1.0 wt%/NaPPDT 1.0 wt% composite gel: 800 Pa, *G*′ for NaPPDT 1.0 wt% gel: 1500 Pa, in the strain sweep, respectively) [66].

**Figure 3.**
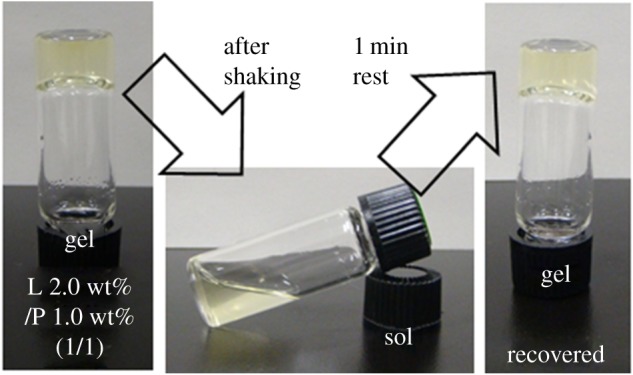
Thixotropic tests of the Laponite/NaPPDT composite hydrogels (denoted as L wt%/ P wt% (1/1, w/w)).

**Figure 4.**
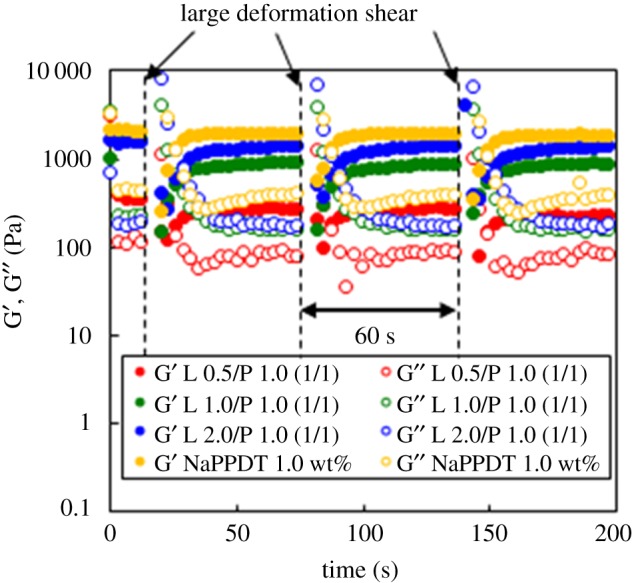
Periodic step-shear test results for the NaPPDT 1.0 wt% hydrogel and Laponite/NaPPDT composite hydrogels (denoted as L wt%/P wt% (1/1, w/w)).

We found that the composite softened while retaining the thixotropic properties, and demonstrated that the mechanical collapse of the composite hydrogel can be recovered after only 1 min of rest (figure 3). This thixotropic behaviour of the composite hydrogel was evaluated quantitatively using the step-shear measurement by applying repeated large deformation forces and periods of rest (figure 4). It was observed that the composite hydrogels in the sol state (*G*′ < *G*′′) were recovered to the gel state (*G*′ > *G*′′). The recovery of the composite hydrogels seemed to be stable above 1.0 wt% Laponite. Furthermore, repeated recovery at each time was observed, indicating that reconstruction of hydrogel after deformation was faster in this composite system. The same thixotropic behaviour was observed in the NaPPDT hydrogels [66], and this may indicate the thixotropic behaviour of the composite hydrogels depending on NaPPDT hydrogel nature.

**Figure 5.**
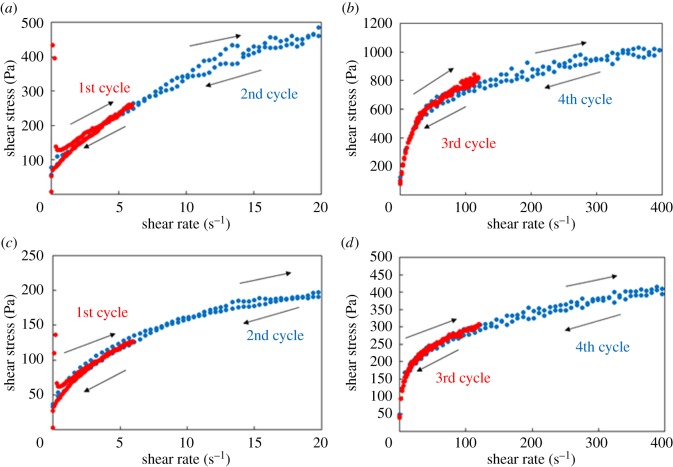
Thixotropic hysteresis loop of the hydrogels. (*a*,*b*) The NaPPDT 1.0 wt% hydrogel. (*c*,*d*) The Laponite 2.0 wt%/NaPPDT 1.0 wt% (1/1, w/w) composite hydrogel.

To further evaluate the thixotropic properties, the thixotropic loop test was undertaken (figure 5) [23,24,80]. As shown in the loops of the NaPPDT hydrogel, the composite hydrogel showed responses with increasing shear rate and corresponding decreased responses with decreasing shear rate. If improved thixotropic behaviour was observed on the hydrogels, the upward and downward curves would coincide (meaning that the breakage and recovery of the gel followed the same curves). Indeed, the thixotropic loops of the composite hydrogel did coincide, indicating that the composite hydrogel had an improved thixotropic nature. Because of this behaviour, this hydrogel might be considered a suitable base material for medicinal products such as ointment-type drug carriers, which require enhanced thixotropy (paintable and spreadable property).

**Figure 6.**
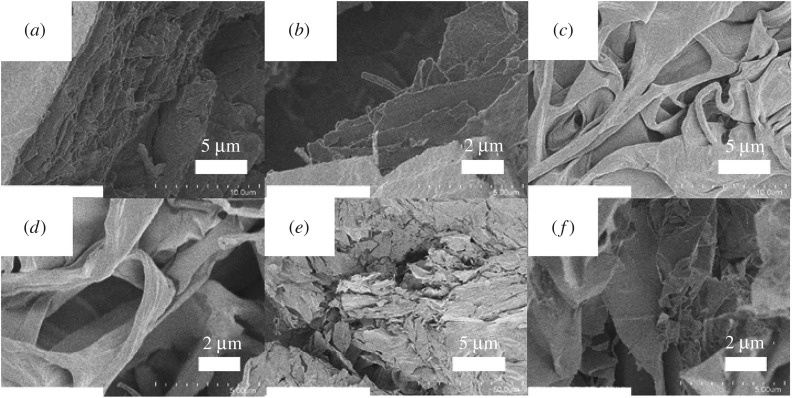
SEM images of dried sample and xerogels: (*a*,*b*) dried sample of 1.0 wt% Laponite, (*c*,*d*) xerogel prepared from the corresponding NaPPDT 1.0 wt % hydrogel, (*e*,*f*) xerogel prepared from the corresponding Laponite 2.0 wt%/NaPPDT 1.0 wt % composite hydrogel.

To investigate the microstructure of the composite hydrogels, scanning electron microscopy (SEM) images of a dried Laponite solution and the corresponding xerogels were obtained (figure 6). However, the individual components of the composite were aggregated (Laponite as silicate discs and NaPPDT as porous, wrinkled networks), and the Laponite 1.0 wt%/NaPPDT 1.0 wt% composite xerogel exhibited a sub-micrometre-scale network structure with a micrometre-scale sheet structure (figure 6). Notably, the composite xerogel contained finer fibres than the NaPPDT xerogel (figure 6*c*,*f*), possibly because of the presence of Laponite, which might prevent aggregation of the NaPPDT polymer chains.

From the electronic supplementary material, figure S2, the SAXS results showed a slope of approximately −2 for the composite gel. This indicates that because of the existence of Laponite the detectable shape of the composite consisted of discs, suggesting that new aggregates capable of forming a new structure were not observed [81,82].

**Figure 7.**
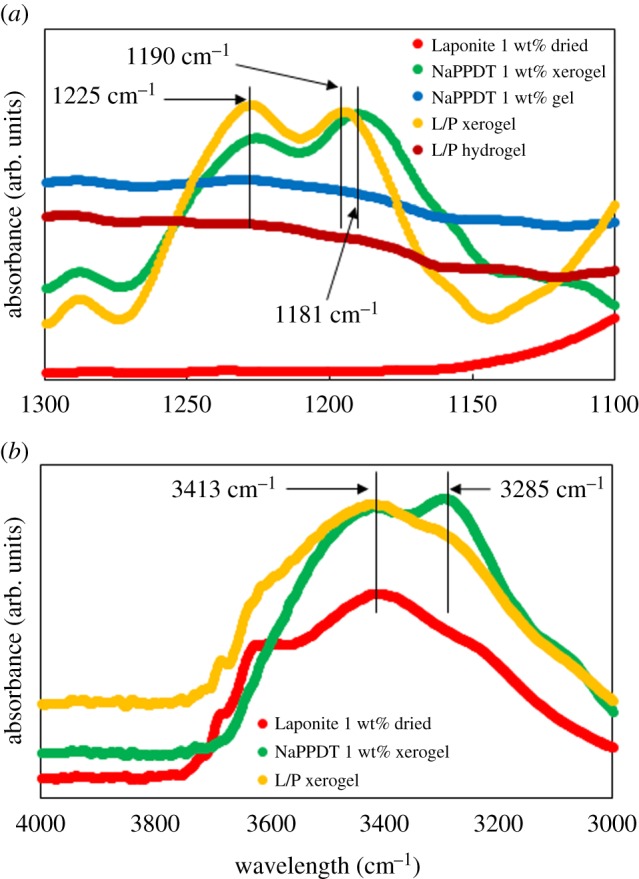
IR spectra of the various aqueous solutions. (*a*) The spectra in the region of sulfonyl stretching, (*b*) the spectra in the region of N-H stretching. L 2.0 wt%/P 1.0 wt% (1/1, w/w) composite hydrogel was denoted as ‘L/P’.

To study the interaction of NaPPDT polymer chains, the infrared (IR) spectrum of the hydrogels and corresponding xerogels in the region of sulfonyl and N-H stretching were measured (figure 7). In figure 7, the absorption peaks of the composite xerogel shifted to a higher wavenumber than those of the composite. This might suggest that the intermolecular interaction between sulfonyl groups and/or N-H groups became weaker in the composite xerogel than in the NaPPDT xerogel. No other shift or change in peaks was seen in the IR spectrum of the composite.

### 3.2. Discussion

A simply mixed molecular gel composed of Laponite and NaPPDT displayed an improvement in the mechanical properties. Although the mixed molecular gel became softer in comparison with the original NaPPDT single gel, the obtained soft gel retained the thixotropic behaviour. This result indicates that the softening of the gel can be compatible with thixotropic behaviour in a mixed gel composed of Laponite and the polymer hydrogelator NaPPDT in the low-concentration region. For uses in paintable base materials of ointments and for applications in medicinal fields [12], this tendency of gels to soften while retaining thixotropic properties upon mixing is thought to be advantageous.

From rheological investigations of the thixotropic behaviour of viscous fluids and gel-like materials, thixotropy is considered to comprise a process that enables reversible breakdown of the three-dimensional network structure of gel-like material into small components, and it is also considered that reconstruction via the reverse process occurs upon shaking or shearing the gel-like material [24]. In this process, small broken components of a material in the sol state should exhibit a response that corresponds to shear thinning of the material. Reversible physical interactions between small broken components play an important role as a driving force for the reversible breakdown and reconstruction (recovery) of the material structure in the thixotropic behaviour of gel-like materials. In organic polymer systems, hydrogen bonds and van der Waals interactions are promising candidates for these reversible interactions between polymer chains in the thixotropic process.

**Figure 8.**
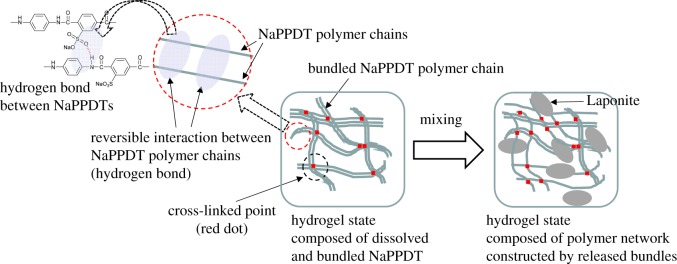
Structural change from the NaPPDT hydrogel to the composite hydrogel by adding Laponite.

As shown above, SEM measurements revealed the presence of both aggregated fibres of NaPPDT in the NaPPDT single gel and a network of finer fibres of NaPPDT in the composite gel. In addition, the suppression of the interactions between NaPPDT polymer chains upon mixing with Laponite was demonstrated by IR measurements. Although the mechanism used to suppress the aggregation of NaPPDT cannot be currently explained, these results suggest the presence of a network of NaPPDT fibres cross-linked by hydrogen bonds in the composite gel that is comparatively less dense than that in the NaPPDT single gel. This change in the density of the fibre network in the composite gel may enable the softening property to be compatible with thixotropy in the low-concentration region on gelation. In this improved network in the composite gel, the number of hydrogen bonds that can participate in the cross-linking of fibres was increased by mixing because the loosening of bundled fibres increases the effective number of hydrogen bonds that are available for cross-linking (figure 8). This increase in the number of hydrogen bonds between NaPPDT chains is thought to be advantageous for the reconstruction of small broken components (small networks), and it contributes to the reversible breakdown and reconstruction of the material during the thixotropic process in the low-concentration region.

On the contrary, an aqueous solution of Laponite with a concentration of 2 wt% is a liquid and becomes a hydrogel when the concentration exceeds 3 wt%. Thus, Laponite tends to form a network and become a gel. It is expected that Laponite should form a small network in an aqueous solution at a lower concentration that is insufficient for the formation of a gel. During the gelation of the composite, the homogeneity of the Laponite network may increase the homogeneity of the NaPPDT network in the composite gel; this may in turn enhance the mechanical properties of the composite gel, as shown by the polymer gel system [83]. Thus, it is speculated that an increase in the number of effective hydrogen bonds between NaPPDT chains and an increase in the homogeneity of the NaPPDT network in the composite gel may play crucial roles in facilitating thixotropic behaviour.

## 4. Conclusion

In conclusion, we demonstrated a mixing-induced softening and thixotropic behaviour of a composite composed of an inorganic nanosheet, Laponite, and a polymer hydrogelator, NaPPDT. While the nanosheet functioned as softening agent in the composite, the nanosheet loosened the polymer bundles and increased the density of the polymer gel network resulting thixotropic behaviour. The mixing-induced softening with retaining of thixotropic behaviour due to the presence of the nanosheet may be applicable to the creation of new soft matter. We are currently investigating and exploring the application of this mixing strategy to other systems to produce composite hydrogels for use as medicinal and healthcare materials.

## Supplementary Material

ESM for figures S1 and S2 (photos under crossed-Nicols and SAXS results)

## Data accessibility

The supporting data for this article have been uploaded as part of the electronic supplementary material.
