# Supplementary material for: New composite thixotropic hydrogel composed of a polymer hydrogelator and a nanosheet
Source: R Soc Open Sci. 2017 Dec 13;4(12):171117. doi: 10.1098/rsos.171117 (PMC5750016; doi:10.1098/rsos.171117)
Supplement: ESM for figures S1 and S2 (photos under crossed-Nicols and SAXS results) [file rsos171117supp1.docx]

**Electronic Supplementary Material**

New Composite Thixotropic Hydrogel Composed of a Polymer Hydrogelator and a Nanosheet

Yutaka Ohsedo,^*1,2,3^ Masashi Oono,^4^ Kowichiro Saruhashi,^4^ Hisayuki Watanabe^3,4^

and Nobuyosih Miyamoto^1^

*^1.^ Department of Life, Environment and Materials Science, Fukuoka Institute of Technology, 3-30-1, Wajiro-Higashi, Higashi-ku, Fukuoka 811-0295, Japan. E-mail: josedo@bene.fit.ac.jp; Fax&Tel: (+81) 92-606-3977.*

*^2.^ Comprehensive Research Organization, Fukuoka Institute of Technology, 3-30-1, Wajiro-Higashi, Higashi-ku, Fukuoka 811-0295, Japan.*

*^3.^ Global Innovation Center, Kyushu University, 6-1 Kasuga-koen Kasuga-city Fukuoka 816-8580, Japan*

^4.^ Nissan Chemical Industries, Ltd., 2-10-1 Tsuboinishi Funabashi Chiba 274-8507, Japan.

**Table of Contents**

**Figures S1 and S2** S2

**References** S2

**Figures S1 and S2**

**
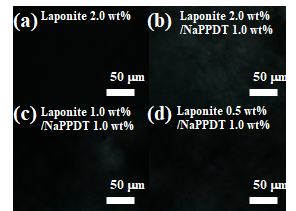
**

**Figure S1.** Laponite/**NaPPDT** composite hydrogels (1/1, w/w) under crossed-Nicols: (a) Laponite 2.0 wt% aq.; (b) Laponite 2.0 wt%/**NaPPDT** 1.0 wt% (1/1); (c) Laponite 1.0 wt%/**NaPPDT** 1.0 wt% (1/1) and (d) Laponite 0.5 wt%/**NaPPDT** 1.0 wt% (1/1).


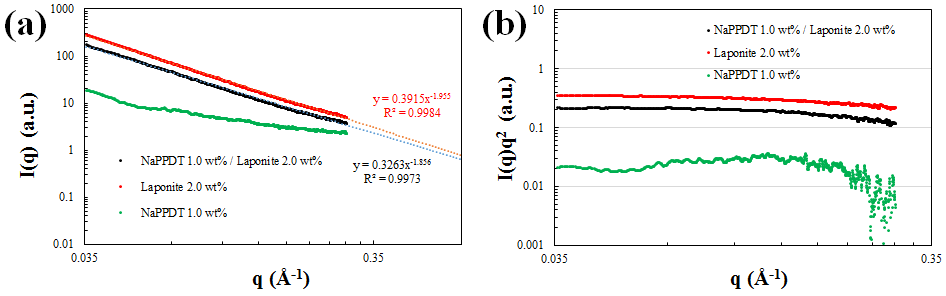


**Figure S2.** SAXS data of Laponite aq. and hydrogels; (a) the composite hydrogel, Laponite aqueous solution and **NaPPDT** hydrogel with exponential approximations and (b) Kratky-type plots of SAXS results. From Fig. S3 (a), the SAXS results showed better exponential fitting of the exponential curve for Laponite aqueous solution (with a slope of ca. –2 in the double logarithmic graph), indicating the existence of flat particles.^1,2^

**References**

1. Glatter O, Kratky O. 1982 *Small angle x-ray scattering*. Academic Press.
2. Bonn D, Kellay H, Tanaka H, Gerard Wegdam, Meunier J. 1999 Laponite: What Is the Difference between a Gel and a Glass? *Langmuir*. **22**, 7534–7536. (doi: 10.1021/LA990167+)
